# Continuing review of ethics in clinical trials: a surveillance study in Iran

**Published:** 2014-12-28

**Authors:** Amin Mohamadi, Fariba Asghari, Arash Rashidian

**Affiliations:** 1 Clinical Research Center, Milad Hospital, and Students Scientific Research Center, Tehran University of Medical Sciences, Tehran, Iran;; 2 Associate Professor, Medical Ethics and History of Medicine Research Center, Tehran University of Medical Sciences, Tehran, Iran;; 3 Associate Professor, Department of Health Management and Economics, School of Public Health, Tehran University of Medical Sciences, Tehran, Iran.

**Keywords:** informed consent, codes of ethics, clinical trial, academic dissertations, Iran

## Abstract

In recent years, notable measures have been taken to protect the rights of participants in biomedical research in Iran. The present study examines possible trends in adherence to ethical codes regarding informed consent after the development of the National Code of Ethics in Biomedical Research (NCEBR) and establishment of research ethics committees.

In this retrospective study, 126 dissertations from Tehran University of Medical Sciences were evaluated for adherence to ethical codes. These dissertations were all in clinical trial design and had been presented in the years 1999 and 2009, that is, precisely before and after the development of the NCEBR.

A checklist was developed to evaluate the ethical issues associated with informed consent. A single investigator retrieved and evaluated the consent forms from the dissertations. Borderline cases were discussed with other investigators to reach a consensus decision. Based on the checklist, the Standardized Ethical Score (SES) was calculated for each consent form. The mean SES and the rate of consent form attachment were compared between the two years.

In total, 70 dissertations had reported obtaining informed consent from study participants, whereas consent forms were attached in only 22 dissertations (17.50%). The percentage of dissertations with the consent form attached increased over time from 12.2% in 1999 to 20.8% in 2009 (*P* > 0.05), but the majority still did not include a consent form. Moreover, the mean SES of consent forms was significantly higher in 1999 (0.746) than in 2009 (0.428), highlighting the need for more training of researchers and improved surveillance by the ethics committees.

A great amount of effort is still needed to make the consent process more ethical, especially for dissertations as a less visible part of academic research. As for students, more systematic training focused on research ethics should be implemented prior to thesis submission.

## Introduction

Medical research involving human subjects has the potential to improve health care and the quality of life for everyone. The study participants, however, may be exposed to a certain degree of risk, and therefore it is the duty of researchers to protect their rights. Furthermore, adherence to ethical codes would safeguard the participants’ rights (1-3).

One of the most basic rights of study participants is autonomy. Since the 1947 Nuremberg Code of Ethics in support of human subjects in medical studies, all published guidelines including the Declaration of Helsinki and the Iranian National Code of Ethics in Biomedical Research (NCEBR) have highlighted the right of autonomy (1-3). One of the most important safeguards protecting participants’ autonomy in biomedical research is the requirement that all researchers obtain voluntary and informed consent from study subjects. With certain exceptions, investigators should obtain a written consent that is signed by the study participants and/or their proxy. In the Declaration of Helsinki, there are seven paragraphs on the conditions and requirements for obtaining consent. Similarly, in the NCEBR, 15 paragraphs are dedicated to the particulars of obtaining research participants’ consent (2, 3). 

Although consent form is required in all human subject experimentations in order to respect participants’ autonomy, obtaining this form without meeting the specific requirements of valid consent gives no assurance of an ethical study. Informed consent requires that patients be given the necessary information (4). However, there is indication that consent forms are not comprehensible for many research participants (5-7). In a prospective study, it was observed that only 20% of the research participants who reviewed consent documents on a physician-patient visit could pass the consent form comprehension test (8). 

Dissertations are more appropriate than articles in scientific journals for evaluating how researchers handle research ethics and how accurately they report ethical issues (9). Unlike scientific journals that usually have ethical guidelines regarding requirements for approval, there are no concrete rules for ethical comments or considerations in dissertations after obtaining protocol approval from Intuitional Review Boards (IRB) (10). In dissertations, researchers have more freedom to report ethical issues comprehensively (11). There is currently not much research regarding ethical issues in dissertations, but existing studies indicate major ethical deficiencies (9, 11-14). A need assessment study in Iran revealed that many medical students and general physicians ignored the importance of research ethics in medical ethics topics (15). 

According to international guidelines for research ethics, all clinical trials should be supervised by a physician adequately experienced in the related field of study (2). Similarly, in Iran the principle investigator of a clinical trial should be an accredited physician on the subject in order to obtain ethical approval. For academic research, students in clinical undergraduate or postgraduate programs can register a dissertation with a clinical trial design if the study is performed under the supervision of a clinical faculty member in the field of study. 

Along with the recent growth in scientific productivity in Iran, some important steps have been taken to protect the rights of participants in biomedical research (3, 16-17). However, no study has examined the content and quality of informed consent forms in Iran to the best of our knowledge. In an early study covering research conducted before 1997 and prior to the development of the NCEBR, investigators reviewed 51 clinical trial dissertations of the Tehran University of Medical Sciences (TUMS). Based on their findings, only one dissertation had reported obtaining informed consents from the participants (14), and none had included an informed consent form (14). 

The present study examines possible trends in adherence to ethical codes regarding informed consent after the development of the NCEBR and establishment of research ethics committees. The results of this study could show how effective the establishment of research ethics committees in Iranian medical schools has been. Although national and international ethical guidelines encompass all types of research with human subjects, we chose clinical trials as the most sensitive type of study in terms of ethical issues. 

## Method

In a retrospective cross-sectional study, we reviewed abstracts of all dissertations approved by three TUMS schools (medicine, pharmacy, and dentistry) in the years 1999 and 2009 to find studies with a clinical trial design. We chose these two years to assess any possible difference in adherence to ethical issues before and after the development of the NCEBR in 2000. We reviewed the Materials and Methods sections of all the dissertations with a clinical trial design to find any claims of obtaining informed consent, and then examined the attached consent forms and information leaflets for research participants in the dissertations. A single investigator examined the forms using a checklist (Table 2). Borderline cases were discussed with other investigators to reach a consensus decision.

We classified the dissertations into undergraduate or postgraduate categories if they were written for a clinical doctorate degree (MD, Pharm D and DDS), or as postgraduate if they pertained to clinical residencies. Additionally, the affiliated department of each dissertation was recorded, but no further information that could identify the authors of the dissertations was retained. Our study protocol was approved by the TUMS research ethics committee. 

**Survey Tool**

In this study, we consulted the NCEBR and two editions of the Declaration of Helsinki (1996 and 2008) in order to extract 14 objective ethical criteria to assess informed consent forms. Four criteria (Table 2, indicators 11-14) were only applicable to dissertations written in 2009 since they were not addressed in former editions of the ethical guidelines. These four criteria were derived from the Iranian ethical guidelines covering clinical trials in 2005, and the 2008 edition of Declaration of Helsinki. A checklist was prepared to review the informed consent forms based on these 14 criteria. Finally, two experts reviewed the checklist for content validity. 

**Analysis**

We calculated an Ethical Score for each consent form where +1 score or 0 score was assigned for each indicator based on whether it was addressed in the consent form or not. The ethical score of each consent form was defined as the sum of these scores. For those indicators that were not relevant to all research studies, the missing data were taken into consideration by standardization of the score. The scores were standardized by dividing the ethical score of each form by the maximum possible score for each consent form. 

Student's t-test was applied for comparison of the mean Standardized Ethical Score (SES) between the years of our study as well as categories of the dissertations. We used one-way Analysis of Variances (ANOVA) to compare the mean SESs among the different schools of TUMS. In order to examine each criterion and the rate of reporting or attaching consent form we used Pearson's chi-square test.

## Results

In the present study, a total of 126 dissertations were reviewed, of which 49 (38.9%) had been approved in 1999 and the rest in 2009. Undergraduate dissertations comprised 54 (42.9%) of the dissertations, and the rest were written for a postgraduate degree (Table 1).

**Table 1:** Categories of dissertations, rate of reporting informed consent and its attachment in the years 1999 and 2009

**Year of ** **approval**	**Undergraduate** **N (%)**	**Postgraduate** **N (%)**	**Reported obtaining ** **consent N (%)** *	**Attached consent ** **form N (%)**
1999	21 (42.9)	28 (57.1)	15 (30.6)	6 (12.2)
2009	33 (42.9)	44 (57.1)	55 (78.6)	16 (20.8)
Total	54 (42.9)	72 (57.1)	70 (55.5)	22 (17.4)

* *A statistically significant difference was observed between the two years*

A total of 70 dissertations had reported obtaining informed consent, while only 22 (17.50%) dissertations included a copy of their consent form. Informed consent was reported in 27 (38.6%) undergraduate and 43 (61.4%) postgraduate dissertations (*P* > 0.05). 

Of the 22 dissertations with attached consent forms, 16 forms (20.8%) were attached to 2009 dissertations and 6 others (12.2%) to 1999 dissertations (*P* > 0.05). Moreover, a total of eight forms (36.4%) were found in the appendix section of undergraduate dissertations. No significant difference was observed in attachment of consent forms across the different categories of dissertations, and none of the reviewed dissertations had included a study information leaflet for their participants.

**Figure 1:**
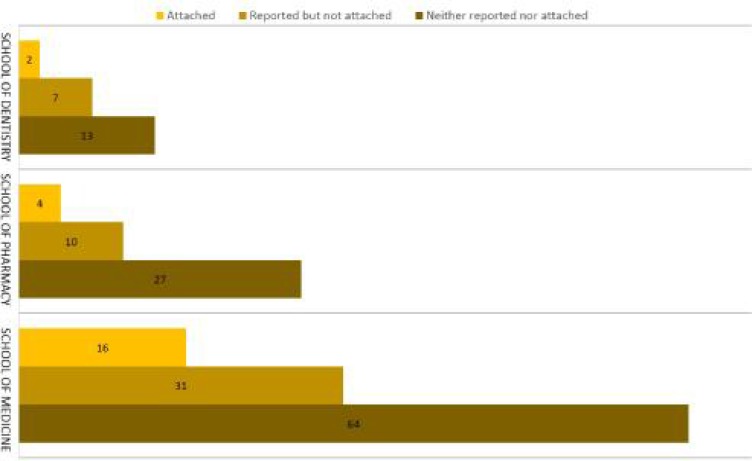
The distribution of dissertations with clinical trial design, reporting consent form or attaching it among three clinical schools of TUMS

Figure 1 shows the distribution of dissertations and rate of reporting or attaching informed consent in the three schools in this study. We observed no significant difference among these schools either in reporting or in attaching informed consent.

In 1999, the mean SES (± Standard Error of Mean) of consent forms was 0.746 (± 0.061), which was significantly higher than 0.428 (± 0.043) in 2009 (*P* = 0.001). The mean SES of consent forms for undergraduate dissertations was 0.459 (± 0.072), which was less than that of postgraduate dissertations at 0.546 (± 0.061) (*P* > 0.05). There was no significant difference in claims of obtaining participants’ consent in dissertations between the two years.

None of the reviewed consent forms met all 14 ethical criteria nor did they declare the source of study funding, investigators’ affiliation, or any possible competing interests. Table 2 shows that of the ten indicators shared between 1999 and 2009, eight were observed more in 1999. Interestingly, although 87% of the 2009 consent forms declared that participant information would be confidential, two dissertations had attached completed and signed consent forms that disclosed participants’ identities. With only one exception, none of the 2009-specific criteria were reported in consent forms.

**Table 2:** Indicators in the two years of dissertation review

**Indicator**		**Year 1999 N (%)**	**Year 2009**	***P*** ** Value**
1	Comprehensible terms and avoidance of using technical terms	6 (100)	14 (87.5)	> 0.999
2	Direct disclosure that participants are enrolled in a research	6 (100)	15 (93.8)	> 0.999
3	Purpose of study	6 (100)	11 (68.8)	0.222
4	Duration of study	5 (83.3)	7 (43.8)	0.162
5	Description of study interventions and other options	3 (50)	5 (31.3)	0.624
6	Anticipated benefits of study	4 (66.7)	3 (18.8)	0.054
7	Potential risks of study	4 (66.7)	4 (25.0)	0.137
8	Confidentiality of participants’ information and possible limitations	2 (33.3)	14 (87.5)	0.025 *
9	Voluntariness of participation in study and the freedom to refuse it	6 (100)	12 (75)	0.541
10	Possibility of allocation to trial or established group (wherever applicable)	2 (40)	5 (55.6)	> 0.999
11	Introducing a contact person or center in case of questions, problems or adverse events	NA	3 (18.3)	NA
12	Investigators’ affiliation	NA	0	NA
13	Funding sources	NA	0	NA
14	Declaration of investigators’ conflicts of interest (or lack thereof)	NA	0	NA

*NA: Not Applicable*

* *A statistically significant difference was observed between the two years *

## Discussion

Respect for the autonomy of medical research participants is one of the basic principles of medical ethics. In order to safeguard this right, all ethical guidelines for biomedical research include directives on obtaining informed consent, conditions that mandate obtaining written consent, and the various features of a consent form. In Iran, numerous measures have been taken in recent years to safeguard the rights of medical research participants (18). These measures include the development of the NCEBR by The Iranian Ministry Of Health and Medical Education in 2000, establishment of local ethics committees in medical schools, and compilation of Specific National Ethical Guidelines for Biomedical Research, which also cover the ethical guidelines for clinical trials, in 2005 (3, 20).

Thus far, only one study has been conducted on the outcome of the above-mentioned measures (21). This study aimed to evaluate the effects of these measures on adherence to ethical guidelines pertaining to consent forms in clinical trial dissertations before and after the development of the NCEBR and establishment of the university ethics committees (18). 

Our findings indicate that during these years, obtaining informed consent in dissertations increased while self-reported adherence to ethical guidelines decreased. However, the quality of consent forms in terms of comprehensibility and disclosure did not improve. It seems that although the rate of obtaining informed consent has increased, the quality of the informed consent forms has not changed. This might be due to the fact that more students were aware of the requirement for informed consent after the development of the NCEBR, but they were not adequately informed of the ethical guidelines. 

Ideally, the SES score for each consent form should be one. A low SES in consent forms can indicate low quality and raise doubts concerning students’ competence in preparing the forms or even their ability to handle ethical issues as independent researchers. Ethics committees failed to address the issue of low SES scores, an oversight that might be due to lack of knowledge among ethics committee members.

Four ethical criteria derived from the Iranian ethical guidelines for clinical trials (2005) and the 2008 edition of the Declaration of Helsinki pertaining to the year 2009 were addressed at the lowest rate in consent forms. These criteria were developed using the 2008 version of the Declaration of Helsinki and were not mentioned in the NCEBR (18). When the NCEBR was prepared in 2000, the earlier versions of the Declaration of Helsinki did not address such ethical issues, and when the Declaration of Helsinki was revised in the following years, no amendment was made to the NCEBR. Nevertheless, the addition of these four criteria cannot account for the lower SES of consent forms in 2009. Firstly, this score is a standardized score, and secondly, the Declaration of Helsinki states that investigators of research studies involving human subjects must consider international ethical, legal, and regulatory norms as well as those of their own country.

Deficiencies in ethical reporting, documentation, and conduct have been noted in other research focusing on students’ dissertations or theses (9 - 13). In a study in Turkey, only 28% of the nurses’ dissertations, which had been approved before 1998, documented obtaining consent from research participants (13). An incremental trend of improvement is observed in the reporting of informed consent in dissertations during the past two decades. For example, the rate of reporting informed consent in Swedish nurses’ dissertations was as low as 0% in 1987 and 30% in 1997, but increased to 85.9% in 2007 (9). A similar trend was also reported in Cameron where none of the reviewed dissertations reported obtaining informed consents in the years before 1990 but the reporting rate increased to 48% in 2010 (11). In Iran, one study showed that only 2% of the dissertations approved between 1994 and 1997 included consent from research participants (14). In the present study, we also noticed an improvement from 30.6% of the dissertations with clinical trial design in 1999 to 78.6% in 2009. 

One of the limitations of this study was that only one stage of the process of obtaining informed consent from research participants was evaluated, and we could not examine the other stages such as physician-patient communication (8). It is also possible that the informed consent attached to the dissertations differed from what was obtained from research participants. In a cohort study, it was stated that authors might report their study data differently than what was submitted to the IRBs (22). It should be noted, however, that the assessment of clinical trial protocols submitted to the IRBs was outside the scope of this study. 

Another limitation of the present study was that it was performed only in one medical school, and therefore the results may not be generalized. On the other hand, the selected university is the largest university in the country and boasts a high rate of scientific productivity (19). We tried to review dissertations from all clinical schools of this university to find all possible clinical trial dissertations. It can be concluded that deficiencies in ethical considerations in the dissertations of this university are a possible red flag for other universities of medical sciences. 

Some of the criteria under investigation were subjective. For instance, an evidence-based judgment is required to evaluate the benefits or hazards of a study. To simplify this judgment, however, when the study investigator included at least one relevant hazard and benefit of the study in consent forms, we assumed that the criterion was addressed. This may have resulted in an over-estimation of adherence, although a major over-estimation is unlikely since few investigators addressed the benefits or hazards of their studies.

Informed consent forms are legal documents that testify to the consent of biomedical research participants, but it has been established that the presence of such forms does not guarantee respect for participants’ autonomy (23). To prove this point, there is indication that consent forms are not comprehensible for the majority of participants in clinical trials (4 - 8).

Unreliable consent forms may be indicative of shortcomings in the process. As a result, we may conclude that much effort is still needed to make the process of obtaining consent more ethical, especially for dissertations as a less visible part of academic research. Students should receive more systematic training focused on research ethics prior to thesis submission, and only those who pass a research ethics course should be permitted to submit a research protocol to the IRB for their thesis. Nevertheless, there is need for further research on the process of obtaining informed consent to offer a more accurate understanding of how this process is addressed in student research and how much attention is being paid by IRBs in reviewing dissertation protocols.
